# A novel ultrasound‐assisted vacuum drying technique for improving drying efficiency and physicochemical properties of *Schisandra chinensis* extract powder

**DOI:** 10.1002/fsn3.2645

**Published:** 2021-11-03

**Authors:** Xuecheng Wang, Shijun Xu, Zhicheng Wu, Yuanhui Li, Yaqi Wang, Zhenfeng Wu, Genhua Zhu, Ming Yang

**Affiliations:** ^1^ Key Laboratory of Modern Preparation of Traditional Chinese Medicine Ministry of Education Jiangxi University of Chinese Medicine Nanchang China; ^2^ State Key Laboratory of Innovative Medicine and High Efficiency and Energy Saving Pharmaceutical Equipment Nanchang China

**Keywords:** drying characteristics, *Schisandra chinensis*, Schisandrol A, ultrasonic power, ultrasound‐assisted vacuum drying

## Abstract

*Schisandra chinensis* (*S. chinensis*) extract powder is an important intermediate for the preparation of many prepared medicines and health products. The physicochemical properties of *S. chinensis* extract powder have been found to vary tremendously and this has been attributed to the long drying time in the traditional drying method. In this study, *S. chinensis* specimens were authenticated as the dry fruit of *S. chinensis* (Turcz.) Baill. *S. chinensis* were extracted twice with 8 L kg^−1^ (liquid to solid ratio) distilled water. The extracts were mixed and concentrated under reduced pressure to 1.24 g cm^−3^. Ultrasound‐assisted vacuum drying (UAVD) was employed as a new approach to improve the efficiency in drying *S. chinensis* extract powder and produce a higher quality product. The effects of drying temperature (70, 80, 90°C), ultrasonic power (40, 120, 200 W), and ultrasonic application time (4, 12, 20 min every 20 min) on the kinetics and quality of *S. chinensis* extract were investigated and compared with the conventional vacuum drying (CVD). It was shown that, with the increase in drying temperature, ultrasonic power, and time of UAVD, the drying time for *S. chinensis* extract to reach the equilibrium moisture decreased. The drying time was reduced by more than 25% when utilizing UAVD compared to the CVD method. The effective moisture diffusivity (*D*_eff_) values for CVD and UAVD were 3.48 × 10^–9^ m^2^·s^–1^ and 7.41 × 10^–9^ m^2^ s^–1^, respectively, at the drying temperature of 80°C, indicating an increase of 112.93%. It was also found that a Weibull distribution model was suitable for predicting the moisture content of *S. chinensis* extract (*R* ^2^ > 0.95). Furthermore, the content of Schisandrol A in the extracts obtained from UAVD was 12.79% higher than that obtained using CVD at 90°C. This demonstrates that UAVD is an efficient drying technique for *S. chinensis* extract.

## Graphical abstract

Ultrasonic enhancement on vacuum drying of Schisandra chinensis extract was studied. The kinetics of process and physicochemical properties were investigated. Ultrasound‐assisted vacuum drying improved the drying efficiency and product quality.

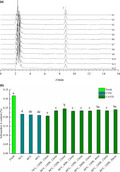

## 1 INTRODUCTION

*S. chinensis* plants are widely distributed all over the world, and China is the country with the most abundant *S. chinensis* resources (Li et al., 2018). The medicinal use of *S. chinensis* fruits is well documented in Chinese, Japanese, Korean, American, Russian and International Pharmacopoeia (Sowndhararajan et al., 2018). In China, dry, mature fruit of *S. chinensis* (Turcz.) Baill. is widely used in clinical practice to improve sleep and immune regulation. The *S. chinensis* fruit is also the potential source of nutrients and contains various chemical constituents (Gao & Wu, 2019; Liu et al., 2017; Wang et al., 2021; Zhang et al., 2020), including lignans, volatile oils, organic acids, terpenoids, flavonoids, and polysaccharides. Previous research reported that *S. chinensis* fruit shows a variety of pharmacological activities. These include: antioxidant, anti‐cough, anti‐hyperprolactinemia, hepatoprotective, anti‐depressant effects, and relieving menopausal symptoms (Hong et al., 2017; Panossian & Brendler, 2020; Yan et al., 2016).

*S. chinensis* extract is a type of brownish powder prepared from *S. chinensis* dried ripe fruit by water extraction, concentration, purification, drying, and smashing. It is an important raw material for many traditional Chinese medicinal formulations, such as Wuweizi granules and Ningshen Buxin tablet. Schisandrol A is one of the most important therapeutic material bases of *S. chinensis* extract due to its significant physiological activities, including antioxidant and sedative‐hypnotic effects (Szopa et al., 2017; Wan et al., 2019). Hence, changes in the main components of *S. chinensis* extract used as raw material during processing should be considered.

Drying methods play a crucial role in the formation of the physicochemical properties of the extract. In the past, the widely used drying method for the *S. chinensis* extract was vacuum drying (VD), which had the advantages of low drying temperature and accommodating a loose product texture. However, the drying efficiency is generally very low (Ozcan‐Sinir et al., 2018). As a result, more effective drying processes are in high demand and ultrasound‐assisted drying was shown promise. The application of ultrasound in the drying process can change the internal microstructure of the material, reduce the water diffusion resistance, and increase the temperature. Because of the synchronous action of cavitation, along with mechanical and thermal effects, heat and mass transfer in the drying process are enhanced. Consequently, the ultrasound‐assisted drying technology has significant advantages of improving the drying efficiency and shortening the drying time and has been widely used in drying food and agricultural products (Fan et al., 2017). Şen and Aydin (2020) investigated the effects of drying air temperature, air velocity, microwave, and ultrasonic power on the drying characteristics of apple slices. The paper reports the application of ultrasonic pressure energy on the apple slices facilitated moisture diffusion and caused a significant increase in the rate of drying. Su et al. (2018) performed an experimental study on the ultrasonic microwave‐assisted vacuum frying of potato chips at low frying temperature. The results indicated that the ultrasonic enhancement reduced the drying time by 20%–28% and decreased the energy used by 20.4%–24.7% compared to microwave‐assisted vacuum frying. All these studies focused on solid materials, but research on drying characteristics and quality of sticky semisolid materials like *S. chinensis* extract has been quite sparse.

Recent studies have shown that the drying temperature, ultrasonic power, and ultrasonic time of UAVD all have an effect on the physical and chemical properties of samples (Li et al., 2020; Zhang & Abatzoglou, 2020). Kroehnke et al. (2018) experimentally investigated the influence of airborne ultrasound‐assisted convective drying on the total color change, water activity, content of carotenoids, and polyphenols of carrots. The results indicated that product improvement occurred with ultrasound as compared to convective drying. However, no information about the influences of the drying method on *S. chinensis* extract was available before this study. Thus, the purpose of this study is to explore the influence of the key process parameters of UAVD on the physical and chemical properties of *S. chinensis* extract, such as moisture content, water activity, hygroscopicity, and Schisandrol A content, and to compare the results with CVD methods.

## 2 MATERIALS AND METHODS

### 2.1 Materials

*S. chinensis* decoction pieces were purchased from the Jiangxi Zhangshu Tianqitang Chinese Herbal Pieces Co., Ltd. Through examination of both macroscopic and microscopic characteristics, all the plant materials were checked and identified as *S. chinensis* by Professor Yang Ming (Jiangxi University of Chinese Medicine). Voucher specimens (S20108) were deposited at the Jiangxi University of Chinese Medicine, Nanchang, Jiangxi province. The chromatographic grade methanol was purchased from Anhui Tiandi high purity solvent Co., Ltd. The analytical grade ethanol was purchased from Xilong Scientific Co., Ltd. Schisandrol A was obtained from Chengdu Chroma‐Biotechnology Co., Ltd. Distilled water was used in all experiments.

### 2.2 Preparation of S. chinensis concentrated solution

**FIGURE 1:**
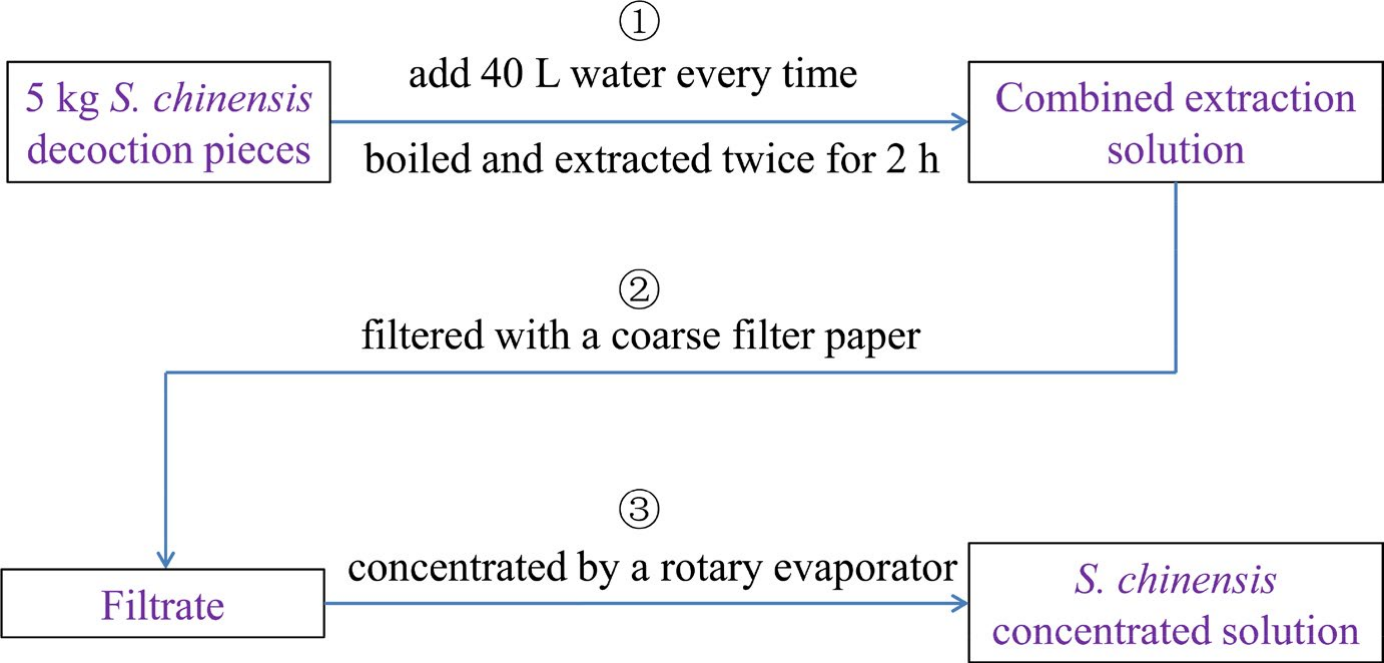
Flow chart of preparation of *S. chinensis* concentrated solution

*S. chinensis* extract was prepared according to the standard method described in the 2020 edition of Chinese Pharmacopoeia (Committee for the Pharmacopoeia of PR China, 2020). According to the process illustrated in Figure 1, *S. chinensis* decoction pieces (5 kg) were immersed in 40‐L water, boiled, and extracted twice at the atmospheric pressure for 2 hr each. The extract was combined and filtered with a coarse filter paper. The filtrate was concentrated to 1.24 g cm^−3^ using a rotary evaporator (R‐1010, Zhengzhou Great Wall Scientific Industrial and Trade Co. Ltd.) at 60°C. The *S. chinensis* extract was finally obtained for subsequent drying.

### 2.3 Drying experiments

#### 2.3.1 CVD

A laboratory‐scale vacuum drying oven (DZF‐6050MBE, Shanghai Boxun Medical Biological Instrument Corp.) was used for the CVD experiments. *S. chinensis* extract (100 g) was placed in a drying tray with a dimension of 150 × 150 × 60 mm. The drying experiments were carried out at 70, 80, 90°C at a constant pressure of −90 kPa.

#### 2.3.2 UAVD

**FIGURE 2:**
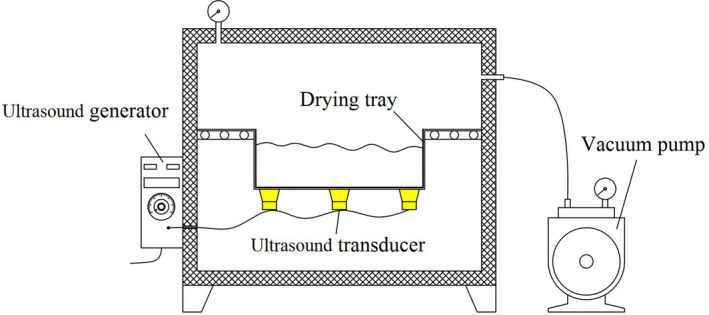
Schematic diagram of the UAVD system

The UAVD equipment used in this study is illustrated in Figure 2. An ultrasound system was incorporated into the CVD equipment, including an ultrasonic generator (THD‐600) and an ultrasonic transducer (50 W, 40 kHz) installed at the bottom of the drying tray. *S. chinensis* extract (100 g) was placed in a drying tray with dimensions of 150 × 150 × 60 mm. The drying experiments were carried out at the drying temperature of 70, 80, 90°C at a pressure of −90 kPa, with an ultrasonic power of 40, 120, 200 W for 4, 12, 20 min every 20 min, respectively.

The *S. chinensis* extract was dried with each drying method until the final moisture content was 0.06 g g^−1^ (dry basis). The moisture content of *S. chinensis* extract samples was determined by removing the container from the dryer. Weighing intervals of 20 min were used during the first 5 hr of processing and then increased to 60 min intervals until the dynamic equilibrium was reached. The weight of the sample was measured using a digital balance (Matou YP20002) with a precision of 0.01 g.

### 2.4 Drying kinetics

The drying process for traditional Chinese medicine extracts is usually described in terms of the change of moisture ratio (MR) with the drying time, and calculated according to Equation (1) (Li, Qi, et al., 2017).

(1) $$\text{MR}=\frac{M_{t}‐M_{e}}{M_{0}‐M_{e}},$$

where *M*_0_, *M_e_*, and *M_t_* are moisture contents (g g^−1^, gram water per gram dry solids) at *t* = 0 min (i.e., initial), equilibrium, and the time instant *t* (min), respectively. Under all experimental conditions in this study, the values of *M_e_* are very close to 0 (i.e., the extracts are almost completely dried); hence, Equation (1) can be simplified as

(2) $$\text{MR}=\frac{M_{t}}{M_{0}},$$

The drying rate (*DR*) was calculated as follows.

(3) $$\text{DR}=\frac{M_{t+\Delta t}‐M_{t}}{\Delta t},$$

where DR is the drying rate (g g^−1^ min^−1^), Δ*t* is the time increment (min), *M_t_*_+Δ_ *_t_* is the moisture content (g g^−1^) at time *t *+ Δ*t* (min).

The Weibull function is often used to describe the moisture change in the drying process of materials, and its expression is given in Equation (4) (Agbede et al., 2020; Karacabey & Buzrul, 2017; Weibull, 1951).

(4) $$\text{MR}=\exp\left[‐{\left(\frac{t}{\alpha }\right)}^{\beta }\right],$$

where *t* is the drying time (min), *α* and *β* are the scale and shape parameters, respectively.

The coefficient of determination (*R* ^2^), root mean square error (*RMSE*), and chi‐square (*χ* ^2^) are the statistical parameters used to evaluate the fitness of the model to the experimental data. If we see that the higher the value of *R* ^2^, the smaller the value of *RMSE* and *χ* ^2^, then the values predicted from the Weibull function are in line with the experimental results and it may be used for predicting these results. The values of *R* ^2^, *RMSE*, and *χ* ^2^ were calculated from Equations (5), (6), and (7), respectively.

(5) $$R^{2}=1‐\frac{\sum_{i=1}^{N}{\left({\text{MR}}_{\exp,i}‐{\text{MR}}_{\text{pre},i}\right)}^{2}}{\sum_{i=1}^{N}{\left({\text{MR}}_{\exp,i}‐\bar{{\text{MR}}_{\text{pre},i}}\right)}^{2}},$$

(6) $$\chi^{2}=\frac{\sum_{i=1}^{N}{\left({\text{MR}}_{\exp,i}‐{\text{MR}}_{\text{pre},i}\right)}^{2}}{N‐Z},$$

(7) $$\text{RMSE}=\sqrt{\frac{1}{N}\sum_{i=1}^{N}{\left({\text{MR}}_{\exp,i}‐{\text{MR}}_{\text{pre},i}\right)}^{2},}$$

where MR_exp,i_, MR_pre,i_, *N*, and *Z* are the moisture ratio obtained experimentally, the predicted moisture ratio, the number of observations, and the number of constants, respectively.

*S. chinensis* extract was spread in a drying tray. The thickness of samples was much less than the length and width. Therefore, according to Fick's second diffusion mathematical model, which is applicable to the plate condition, the effective diffusion coefficient of water in the drying process can be calculated from Equation (8) (Liu et al., 2018).

(8) $$\text{MR}=\frac{8}{\pi^{2}}\exp\left(‐\pi^{2}\frac{D_{\text{eff}}t}{4L^{2}}\right),$$

where *D*_eff_ was the effective moisture diffusivity (m^2^ s^−1^), *t* was drying time (s), and *L* was the half‐thickness of samples (m). By transforming Equation (8), the linear Equation (9) between the natural logarithm of MR (ln MR) and drying time *t* (s) can be obtained.

(9) $$\ln\text{MR}=\ln\frac{8}{\pi^{2}}‐\pi^{2}\frac{D_{\text{eff}}t}{4L^{2}},$$

### 2.5 Physical and chemical properties

#### 2.5.1 Sample preparation

*S. chinensis* extract dried under different conditions was crushed and made into powder using a laboratory‐scale grinder (XY‐200, Zhejiang Yongkang Songqing Hardware Factory). Then it was sifted using an 80 mesh siever (S49–200, Xinxiang Gaofu Machinery Co., Ltd.) and stored in a glass desiccator at room temperature.

#### 2.5.2 Moisture content and water activity

The moisture content of dried samples was determined using the oven method (Ran et al., 2019). Samples were dried in the oven for 5 hr at 105 ± 1°C to achieve a constant weight. The water activity was measured using a water activity meter (HD‐6, Wuxi Huake Instrument Co., Ltd).

#### 2.5.3 Hygroscopicity

Hygroscopicity was determined by adopting the method proposed by Gallo et al. (2011). Approximately 2 g of dried samples were stored in containers at 25°C and 75.29% relative humidity (RH). The RH in the containers was modulated by the use of the saturated aqueous solution of NaCl. The samples were precisely weighed at the 120‐hr mark. The hygroscopic rate was calculated according to Equation (10) and the hygroscopic curve was drawn.

(10) $$W_{m}=\frac{m_{t}‐m_{0}}{m_{0}}\times 100\%,$$

where *W*_m_ is the hydroscopic ratio, *m*_t_ and *m*_0_ are the final and the initial mass of samples (g).

#### 2.5.4 Water solubility

Water solubility was determined according to the method reported in Salahi et al. (2017). Hundred milliliters of distilled water were transferred into a blender jar (MS‐S BlueSpin, Zhengzhou Nanbei Instrument Equipment Co., Ltd.), and 1 g of powder sample was carefully added to the blender and mixed at 500 rpm for 5 min. The solution was transferred in a tube and centrifuged (H2050R, Hunan Xiangyi Laboratory Instrument Development Co., Ltd.) at 10,000 rpm for 5 min. Next, an aliquot of the supernatant (25 ml) was transferred into pre‐weighed Petri dishes and oven‐dried at 105°C to a constant weight. The solubility (%) was calculated as the proportion of the mass of solids in Petri dishes to the total mass (1 g).

#### 2.5.5 Determination of Schisandrol A content

High‐performance liquid chromatography (HPLC) analysis was performed using an Agilent 1260 series high‐performance liquid chromatography system equipped with a G1314–A pump, an automatic injector, and a diode array detector using the same method as reported in Chen et al. (2018). The mobile phase was methanol: water (65:35) with a flow rate of 1.0 ml min^−1^. The wavelength used to detect Schisandrol A was set at 250 nm. A calibration curve based on Schisandrol A standard solutions showed good linearity over the range of 0.018–0.048 mg ml^−1^. The regression equation of Schisandrol A was *Y* = 19,508.57*X* + 11.35 (*R* ^2^ = 0.998, *n* = 6), where *Y* is the peak area of Schisandrol A and X is the concentration of Schisandrol A (mg ml^−1^). The accuracy of the method was evaluated from the results of the recovery test. The content of the Schisandrol A standard was determined by adding equal amounts of Schisandrol A into six sample solutions of known concentrations. The average recovery was 100.14%. RSD of the relative peak areas of Schisandrol A was 0.50%, less than 1%. The repeatability of the HPLC method was determined by analyzing a random sample in sextuplicate. RSD of the relative peak areas of Schisandrol A was 1.22%, less than 2%. The stability of the HPLC method was measured by analyzing a sample every 3 hr for 24 hr. RSD of the relative peak areas of Schisandrol A was 1.15%, less than 2%. The above results demonstrate that the linearity, recovery rate, repeatability, and stability of the HPLC method were satisfactory.

### 2.6 Statistical analysis

The results were statistically evaluated by one‐way analysis of variance (ANOVA) using MATLAB (version R2019a, MathWorks, Inc.) and OriginPro (version 8.5.1, OriginLab, Inc.). *p* < .05 was defined as statistically significant. All drying experiments were conducted in triplicate, and the values obtained from these experiments were averaged.

## 3 RESULTS AND DISCUSSION

### 3.1 Drying characteristics

**FIGURE 3:**
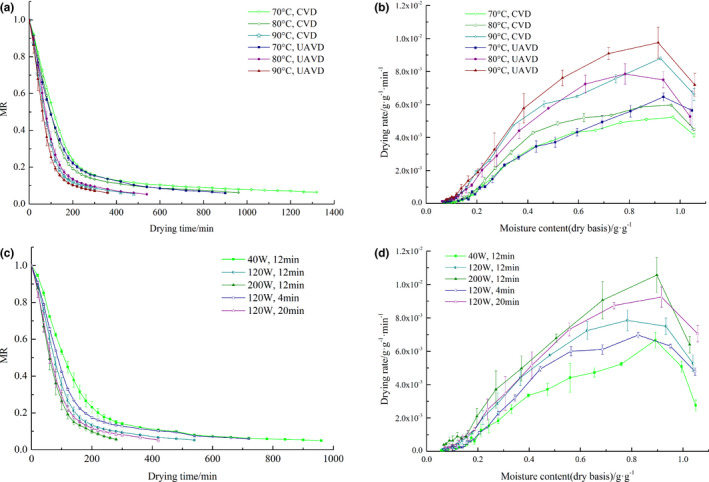
Drying characteristics under different conditions for the *S. chinensis* extract. (a) Drying curves at different temperatures of CVD and UAVD; (b) Drying rate curves at different temperatures of CVD and UAVD; (c) Drying curves with different ultrasonic power and frequency of UAVD; (d) Drying rate curves with different ultrasonic power and frequency of UAVD

It is well‐recognized in the literature that UAVD combines the advantages of ultrasonic and vacuum drying because it can promote water diffusion and evaporation in the drying process. Efficient drying of materials under low‐temperature conditions is feasible (Souza da Silva et al., 2019; Edvaldo et al., 2018). The CVD and UAVD drying characteristic curves under different conditions are shown in Figure 3. The changes in moisture content and drying rate under different drying conditions are also shown. The results shown in Figure 3a indicate that the drying curves for the same drying method under different drying temperatures have similar trends. The time required to reach the equilibrium moisture content of 0.06 g g^−1^ decreased as the temperature increased. The drying times for CVD were 1,320, 960, and 480 min at 70, 80, and 90°C, respectively. The drying times were shortened to 900, 540, and 360 min with an ultrasonic power of 120 W and treatment time of 12 min, reducing 31.82%, 43.75%, and 25%, respectively.

Figure 3b shows the evolution of drying rates at different temperatures for CVD and UAVD of the *S. chinensis* extract. The drying rates mainly decreased with decreasing moisture contents in all drying processes. The average drying rates of CVD and UAVD were 1.45 × 10^−3^, 1.80 × 10^−3^, 2.71 × 10^−3^ g g^−1^ min^−1^, and 1.84 × 10^–3^, 2.51 × 10^–3^, 3.08 × 10^–3^ g g^−1^ min^−1^ at the drying temperature of 70, 80, and 90°C, respectively. The drying rate was effectively increased once the ultrasound was applied (*p* < .05), indicating that the ultrasound could improve the internal mass transfer rate by reducing the internal diffusion resistance. In addition, the mechanical effect of ultrasonic wave propagation in the liquid medium could produce a powerful shear force. The ultrasound cavitation effect of UAVD produced local transient high temperature and high pressure inside the material. Furthermore, the collapse of the cavitation bubble produced powerful shock waves, which in turn produced micro jets and acoustic impact at the solid‐liquid interface. These two effects increased the turbulence amplification of the water diffusion channel in the extract and thus improved the water migration ability (Chemat et al., 2011; Chen et al., 2019; Garcia‐Perez et al., 2012). Tekin and Baslar (2018) also found that the UAVD drying for red peppers shortened the drying period by 25% and increased the effective moisture diffusivity by 89%, compared to CVD.

The drying curves of *S. chinensis* extract of UAVD at different ultrasonic power levels and times were shown in Figure 3c. With an ultrasonic power of 40 W, the time required to reach the equilibrium moisture content was 960 min. The power increased to 120 and 200 W, the drying time was decreased from 540 to 280 min, which was 43.75% and 70.83% less, respectively. The time required to reach the equilibrium moisture content was 720 min when ultrasound was applied for 4 min every 20 min. As the ultrasound time was extended to 12 and 20 min, the drying time was decreased from 720 min to 540 and 420 min, shortened by about 25% and 41.67%, respectively. As can be seen from Figure 3d, with an ultrasonic power of 40, 120, and 200 W, the average drying rate of *S. chinensis* extract was 1.79 × 10^–3^, 2.51 × 10^–3^, and 3.46 × 10^–3^ g g^−1^ min^−1^, respectively. The average drying rate of the *S. chinensis* extract was 2.13 × 10^–3^, 2.51 × 10^–3^, and 2.88 × 10^–3^ g g^−1^ min^−1^ with ultrasound times of 4, 12, and 20 min, respectively. In fact, with the increase of ultrasonic power and time, the mechanical and cavitation effects of ultrasound become stronger. Therefore, we concluded that higher ultrasonic power was beneficial to increase the turbulence and fluidity of water in the material and weaken the adsorption force between water and tissue (Jiang et al., 2020). Subsequently, the mass transfer rate could be improved and the drying time could be shortened significantly.

### 3.2 Drying kinetics

The model parameters *α* and *β* of the Weibull function are correlated with the drying process parameters, which is useful to enhance the understanding of the drying mechanisms (Deng et al., 2020). Therefore, the Weibull function is always used to describe the drying process. Coefficients of the Weibull function and the comparison criteria for evaluating the fitting quality, including *R* ^2^, *RMSE*, and *χ* ^2^ are given in Table 1. The *R* ^2^ were higher than 0.95 for all drying conditions, and the *χ* ^2^ and *RMSE* values for the modeling were very low (<0.0033 and <0.0557, respectively). This indicates that the Weibull function could well describe the drying of the *S. chinensis* extract in this study. As the temperature increased from 70°C to 90°C, the CVD value decreased from 161.95 min to 96.93 min, and the corresponding UAVD value decreased from 144.21 min to 85.23 min. Similar behaviors were also observed during the CVD of maqui berries (Issis et al., 2019) and ginger slices (Wang, Bai, et al., 2019). Therefore, whether using CVD or UAVD, increasing the drying temperature could shorten the drying time, as expected. The scale parameter *α* represents the time needed to accomplish approximately 63% of the process (Corzo et al., 2008). The *α* values of UAVD were all lower than that of CVD at the same temperature, indicating that ultrasonic‐assisted vacuum drying could effectively improve the drying efficiency. At the same drying temperature, the *α* value was also reduced with the increasing ultrasonic power and time, which was consistent with the observation of the drying rates (see Table 1).

**TABLE 1:** Weibull fitness parameters and *D*_eff_ for various drying experiments considered

Drying conditions	*α* (min)	*β*	*R* ^2^	*χ* ^2^	*RMSE*	*D*_eff_ (m^2^ s^−1^)
CVD						
70°C	161.95	0.92	0.9547	0.0033	0.0557	2.42 × 10^–9^
80°C	138.01	1.00	0.9641	0.0028	0.0509	3.48 × 10^–9^
90°C	96.93	1.05	0.9824	0.0015	0.0368	8.31 × 10^–9^
UAVD						
70°C, 120 W, 12 min	144.21	0.86	0.9763	0.0018	0.0402	3.89 × 10^–9^
80°C, 120 W, 12 min	105.21	1.08	0.9799	0.0018	0.0397	7.41 × 10^–9^
90°C, 120 W, 12 min	85.23	1.07	0.9825	0.0016	0.0371	1.10 × 10^–8^
80°C, 40 W, 12 min	153.49	1.07	0.9775	0.0019	0.0423	4.06 × 10^–9^
80°C, 200 W, 12 min	84.09	1.12	0.9894	0.0010	0.0298	1.40 × 10^–8^
80°C, 120 W, 4 min	124.39	0.99	0.9699	0.0024	0.0472	4.96 × 10^–9^
80°C, 120 W, 20 min	91.18	1.02	0.9833	0.0014	0.0356	9.59 × 10^–9^

The shape parameter *β* of the drying model varied from 0.86 to 1.12 for different drying conditions. The shape parameter *β* has almost no effect by ultrasonic power and time. Similar values were found for drying coroba slices (Corzo et al., 2008) and vacuum far‐infrared‐dried Rehmanniae (Liu et al., 2016). The variation of *β* values could be induced with various mechanisms, such as diffusion, convection, and relaxation. When *β* = 1, the Weibull distribution reduces to the first‐order kinetics.

*D*_eff_ is an important parameter to characterize the drying efficiency, especially in the falling‐rate drying period (Minaei et al., 2012). *D*_eff_ values for various drying experiments considered were presented in Table 1. At the same drying temperature, the *D*_eff_ value of UAVD was higher than that of CVD. The water effective coefficient increased and the drying rate accelerated with the increase in temperature, ultrasonic power, and time, which was consistent with the previous studies (Jiang et al., 2020; Tekin & Baslar, 2018). Furthermore, the variation trend of effective water diffusion coefficient under different drying conditions was consistent with the drying curve, which again confirms the influence of temperature, power, and time on the UAVD process.

### 3.3 Physical and chemical properties

#### 3.3.1 Moisture content and water activity

Moisture content is an important property of *S. chinensis* extract powder, which affects its flowability and storage stability. The value of water activity is also directly related to the growth rate of microorganisms. Different water activity in samples leads to variation in storage life. The growth of microorganisms can be controlled by monitoring water activity. Therefore, water activity has gradually become an important index in food, cosmetics, pharmaceuticals, biological products, and other industries (Chitrakar et al., 2019). From Table 2, the moisture content and water activity of UAVD‐dried samples varied from 9.75% to 11.82% and 0.252 to 0.289, respectively. In this study, the range of moisture content and water activity of UAVD‐dried samples were similar to CVD‐dried samples. The moisture content of samples varied significantly (*p* < .05) with the drying method at the same temperature. From the results, the water activities of the samples were less than 0.35, meaning that all samples hold relative stability in terms of both microbiology and biochemistry (Carpin et al., 2016).

**TABLE 2:** Moisture content, water activity, hygroscopicity, and water solubility of *S. chinensis extract* powder

Drying method	Moisture content (%)	Water activity	Hygroscopicity (g of adsorbed moisture per 100 g of sample)	Water solubility (%)
CVD				
70°C	11.33 ± 0.17^b^	0.287 ± 0.0025^ab^	20.58 ± 1.63^b^	85.48 ± 0.08^cde^
80°C	10.41 ± 0.04^e^	0.254 ± 0.0006^d^	23.09 ± 0.40^a^	85.48 ± 0.33^cde^
90°C	10.36 ± 0.02^e^	0.241 ± 0.0061^e^	22.87 ± 0.17^a^	86.33 ± 0.28^bc^
UAVD				
70°C, 120 W, 12 min	11.82 ± 0.14^a^	0.289 ± 0.0020^a^	22.15 ± 0.07^ab^	84.87 ± 0.03^de^
80°C, 120 W,12 min	11.65 ± 0.01^a^	0.268 ± 0.0006^c^	23.60 ± 0.03^a^	85.20 ± 0.80^de^
90°C, 120 W, 12 min	10.71 ± 0.11^d^	0.254 ± 0.0006^d^	22.14 ± 0.45^ab^	85.25 ± 0.40^de^
80°C, 40 W, 12 min	11.03 ± 0.04^c^	0.265 ± 0.0010^c^	22.45 ± 0.40^a^	86.38 ± 0.23^b^
80°C, 200 W, 12 min	11.44 ± 0.00^b^	0.281 ± 0.0025^b^	23.43 ± 0.21^a^	85.65 ± 0.10^bcd^
80°C, 120 W, 4 min	11.30 ± 0.08^b^	0.271 ± 0.0006^c^	22.07 ± 0.67^ab^	84.68 ± 0.03^e^
80°C, 120 W, 20 min	9.75 ± 0.02^f^	0.252 ± 0.0015^d^	22.20 ± 0.40^ab^	87.45 ± 0.20^a^

Note

Values are expressed as mean ± *SD* of three replicated determinations. Means with the different letter in the same column are significantly different (*p* < .05).

#### 3.3.2 Hygroscopicity

Hygroscopicity refers to the ability of the material to absorb water from the environment. It is an important performance marker in the production process for traditional Chinese medicines and affects the properties and stability of the extract powder (Gallo et al., 2015). The hygroscopic properties of the samples under different drying conditions were also presented in Table 2, and the maximum moisture absorption ranged from 20.58% to 23.60%. The results of ANOVA showed that the drying mode and the drying conditions (power, temperature, ultrasonic time) during UAVD had no significant difference in the hygroscopic properties (*p* > .05). The microstructure formed during the drying process of the extract determines the hygroscopicity of the dried powder, so it can be inferred the *S. chinensis* extract powders produced by the UAVD method have similar microstructures to that obtained from CVD.

#### 3.3.3 Water solubility

Water solubility is an index to evaluate the affinity between the extract powder and the aqueous solution, and directly affects the efficacy of drugs and healthy food (Jafari et al., 2017). As shown in Table 2, the solubility of CVD samples was close to that of UAVD samples. There were no significant differences among the solubility (85.48 ± 0.08%, 85.48 ± 0.33%, 86.33 ± 0.28%) of vacuum‐dried powders obtained at three different temperatures (70°C, 80°C, 90°C) with *p* > .05. This is consistent with the results of Li et al., 2017b. For UAVD, the dissolution of samples showed no significant correlation (*p* > .05) at different temperatures and ultrasonic power. However, the dissolution of samples increased significantly with the extension of ultrasonic time (*p* < .05). The increase in ultrasonic time can not only reduce the total drying time, it can also increase the solubility through the formation of internal cellular structure. This was in agreement with the result of Wang et al., 2019a.

#### 3.3.4 Schisandrol A content

**FIGURE 4:**
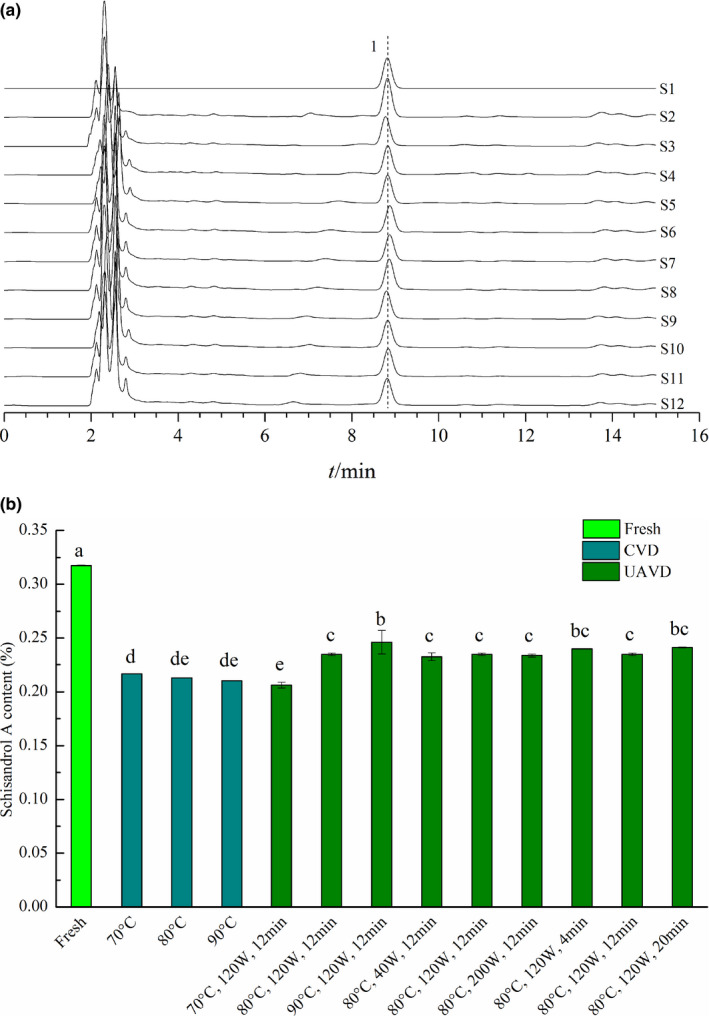
The effects of different drying conditions on Schisandrol A content of the *S. chinensis* extract powders. (a) HPLC chromatogram. S1: Schisandrol A standard; S2: Fresh; S3:70°C, CVD; S4: 80°C, CVD; S5: 90°C, CVD; S6:70°C, 120 W, 12 min, UAVD; S7: 80°C, 120 W, 12 min, UAVD; S8: 90°C, 120 W, 12 min, UAVD; S9: 80°C, 40 W, 12 min, UAVD; S10: 80°C, 200 W, 12 min, UAVD; S11: 80°C, 120 W, 4 min, UAVD; S12: 80°C, 120 W, 20 min, UAVD;1: Schisandrol A; (b) Schisandrol A content. Bars that display different letters are shown as significantly different (*p* < .05)

The effects of different drying conditions on Schisandrol A content of the *S. chinensis* extract powders are displayed in Figure 4. As shown in Figure 4a, the retention time of Schisandrol A was about 8.8 min based on the HPLC measurements in this study. According to the experimental results presented in Figure 4b, the Schisandrol A content varied significantly with the drying method (*p* < .05). Before drying, the Schisandrol A content of fresh concentrate was 0.318%. The results showed that dried samples had a lower Schisandrol A content than the fresh concentrate. The Schisandrol A content was 0.217%, 0.213%, and 0.210%, respectively, with a CVD temperature of 70, 80, and 90°C. We observed that the Schisandrol A content decreased slightly with the increase in temperature, but the influence was not significant (*p* > .05). The content of Schisandrol A in UAVD samples was generally higher than that in the CVD samples under all drying conditions except 70°C, 120 W, 12 min. The observed lowest content of Schisandrol A at 70°C of UAVD‐dried samples could be attributed to the longest total treatment time by 120 W ultrasonic power. The stability of Schisandrol A may be affected by a long time and high power ultrasonic treatment. At the drying temperature of 90°C, the content of Schisandrol A in the UAVD and CVD samples was 0.247% and 0.219%, respectively, i.e. 12.79% higher in the UAVD samples. In the UAVD process, the effects of ultrasonic power and time on the Schisandrol A content of *S. chinensis* extract were not significant (*p* > .05). However, with the increase in drying temperature, the content of Schisandrol A increased significantly (*p* < .05). This is due to the fact that the UAVD time is significantly shortened at a higher drying temperature, which increases the retention rate of Schisandrol A (Tekin et al., 2017).

## 4 CONCLUSIONS

In this study, UAVD was employed to dry *S. chinensis* extract. Then, the UAVD drying kinetics and physicochemical properties of the *S. chinensis* extract were examined and compared with CVD. The Weibull model was used to describe the drying processes. To better understand the UAVD method involved in the drying of *S. chinensis* extract, the influences of drying temperature, ultrasonic power, and time were evaluated. The results showed that the drying time was reduced by more than 25% with UAVD. Moreover, the content of Schisandrol A in the UAVD samples was 12.79% higher than that obtained in CVD at the drying temperature of 90°C. Therefore, UAVD is a better alternative drying technique for *S. chinensis* extract, as it can reduce the drying time while maintaining the physicochemical properties.

## CONFLICT OF INTERESTS

The author declares no conflict of interests.

## AUTHOR CONTRIBUTIONS

**Shijun Xu:** Data curation (equal). **Zhicheng Wu:** Formal analysis (equal). **Yuanhui Li:** Methodology (equal). **Yaqi Wang:** Software (equal). **Zhenfeng Wu:** Conceptualization (equal); Writing‐review & editing (equal). **Genhua Zhu:** Supervision (equal); Writing‐review & editing (equal). **Ming Yang:** Supervision (equal).

## ETHICAL APPROVAL

This study does not involve any human or animal testing.

## Data Availability

The data that support the findings of this study are available from the corresponding author upon reasonable request.
